# Defining the Sequence Elements and Candidate Genes for the *Coloboma* Mutation

**DOI:** 10.1371/journal.pone.0060267

**Published:** 2013-04-09

**Authors:** Elizabeth A. Robb, Parker B. Antin, Mary E. Delany

**Affiliations:** 1 Department of Animal Science, University of California Davis, Davis, California, United States of America; 2 Department of Molecular and Cellular Medicine, University of Arizona, Tucson, Arizona, United States of America; Temasek Life Sciences Laboratory, Singapore

## Abstract

The chicken *coloboma* mutation exhibits features similar to human congenital developmental malformations such as ocular coloboma, cleft-palate, dwarfism, and polydactyly. The *coloboma*-associated region and encoded genes were investigated using advanced genomic, genetic, and gene expression technologies. Initially, the mutation was linked to a 990 kb region encoding 11 genes; the application of the genetic and genomic tools led to a reduction of the linked region to 176 kb and the elimination of 7 genes. Furthermore, bioinformatics analyses of capture array-next generation sequence data identified genetic elements including SNPs, insertions, deletions, gaps, chromosomal rearrangements, and miRNA binding sites within the introgressed causative region relative to the reference genome sequence. *Coloboma*-specific variants within exons, UTRs, and splice sites were studied for their contribution to the mutant phenotype. Our compiled results suggest three genes for future studies. The three candidate genes, *SLC30A5* (a zinc transporter), *CENPH* (a centromere protein), and *CDK7* (a cyclin-dependent kinase), are differentially expressed (compared to normal embryos) at stages and in tissues affected by the *coloboma* mutation. Of these genes, two (*SLC30A5* and *CENPH*) are considered high-priority candidate based upon studies in other vertebrate model systems.

## Introduction

Three percent of the four million children born each year in the United States have a birth defect [1], [2]. Of those congenital defects, ∼50% are due to genetic causes of which 30% are heritable [2]. The UCD-Coloboma.003 (Co.003) chicken congenic line is an important animal biomedical model as it expresses features similar to human congenital defects including cleft palate and dwarfism, and eye, limb, digit, and visceral abnormalities [3], [4]. Thus, identification of the underlying genetic mechanism causing the chicken coloboma phenotype provides a unique opportunity to elucidate the cause of similar multisystem syndromes in human. The classic chick embryo model (see Stern [5]), with its *in ovo* easy access, provides great value in this regard [6]–[10]. The utility of the chick embryo model was further improved by the availability of the chicken reference genome sequence [11], advanced tools and high-throughput technologies, as well as the existence of well-phenotyped developmental mutations [4], [12]–[14]. Such advantages led to the National Institute of Health recognition of the chicken as a model organism for biomedical research (http://www.nih.gov/science/models/gallus/).

A key characteristic of the UCD-Co.003 genetic line, ocular coloboma, i.e., reduction of tissue near and around (e.g., eyelid) or in structures of the eye (e.g., lens, macula, optic nerve, uvea), is present in ∼1 per 10,000 human births [15] and accounts for up to 10% of childhood blindness [16]. Congential coloboma was first reported in human in 1870 ([17] and references therein) and in the chicken in 1958 [18] and has been described in both organisms as an individual malformation or occurring in conjunction with a number of other multisystem syndrome abnormalities (e.g., craniofacial, skeletal, limb, and urogenital) and/or other ocular defects [3], [4], [12], [17]–[20]. Although most cases of human coloboma are considered sporadic, sex-linked, autosomal recessive, and autosomal dominant modes of inheritance have been reported [21]. Of such, approximately a dozen genes have been associated with the coloboma defect [22]–[31] but account for only a small subset of reported cases of coloboma, thus research using animal models is essential to understand the etiology of the condition. The inherited malformations (i.e., coloboma of the eye, cleft-palate, dwarfism, truncated limbs, exposed viscera, the addition or loss of a digit on the feet) of the UCD-Co.003 genetic line provides the unique opportunity to uncover the genes and pathways involved in coloboma as well as the other associated malformations described above. Such comparative vertebrate knowledge will contribute to a greater understanding of genes involved in human development as well as improved knowledge of shared vertebrate developmental pathways.

Previously we reported on the chromosomal mapping and size of the causative (linked) region (CR) for the *coloboma* mutation using a 60****K SNP array [4]. The discovered molecular markers were then used in new individuals to identify recombination events; this in turn further reduced the size of the linked region. Subsequently, RNA-bait sequence probes, complementary to the reduced 990 kb region, were generated for use in next-generation sequencing (NGS) of the region. That is, a targeted genomic capture enrichment technology (a.k.a. capture array (CA)) was employed to identify the element causing this mutation [32]. Here we focus on the CA/NGS analysis of the genetic features discovered (e.g., SNPs, insertions, deletions) which include normal polymorphisms of the introgressed region as well as the mutant-specific variant element. Our goal was to discriminate between the two categories of variants within the region to find high priority candidates for future study. The Co.003-specific variant elements were evaluated for their contribution to the mutant phenotype using a variety of genetic, genomic, and bioinformatics techniques (e.g., sequence verification, splice site analysis, miRNA binding sites, etc.). Additionally, putative translocation events in the Co.003 genetic line, identified through genomic alignments, were assessed for their legitimacy. Finally, a set of genes encoded in the minimum causative region (176 kb) were studied for their expression in both mutant and normal embryos during several stages of early embryogenesis by whole-mount *in situ* hybridization (ISH) to evaluate their potential involvement. Combined, these studies reveal three high-priority candidates for future functional analysis.

## Methods

### Ethics Statement

Animals used for the study were under the care and supervision of trained staff and as per protocols approved by the University of California, Davis Institutional Animal Care and Use Committee.

### Genetic Lines

**Figure 1:**
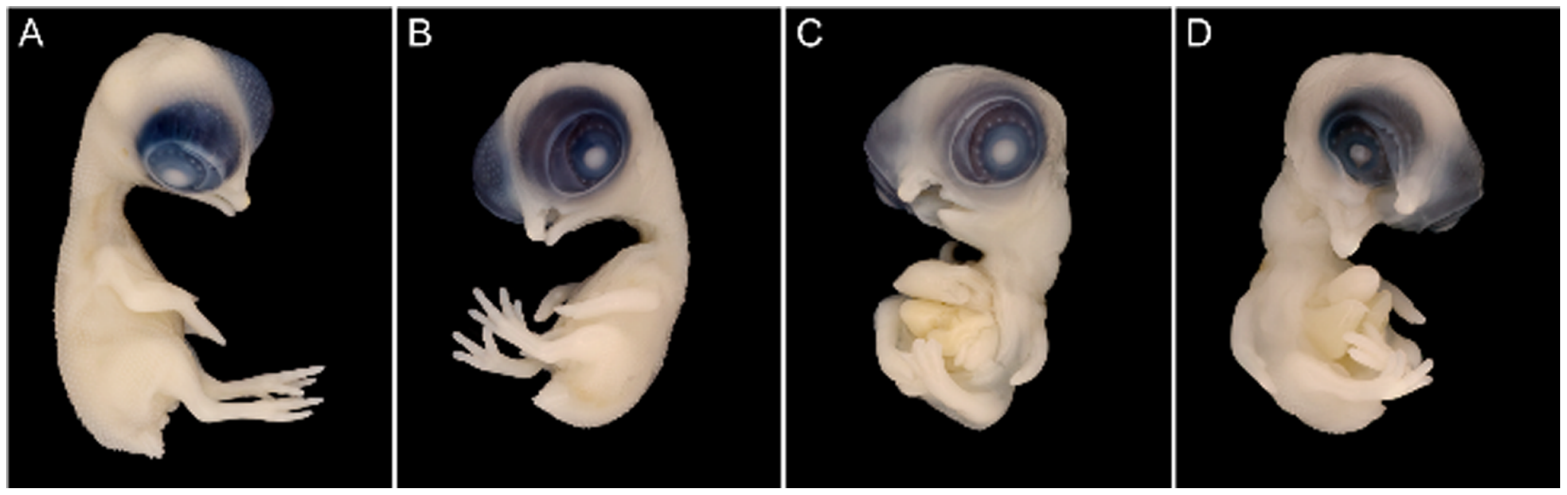
Phenotype variability among UCD-Coloboma.003 mutant embryos. (**A**) Normal, non-carrier (Z^+^/Z^+^) UCD-Coloboma.003 chicken embryo. (**B**) *Mild expression.* UCD-Coloboma.003 mutant embryo (Z^co^/W) displaying mild truncation of the limbs, mild cleft-palate, mild dwarfism, mild exposed viscera, oligodactyly (loss of digit - three toes displayed with the loss of the hallux, compared to the normal four toes, termed anisodactyly) on the embryo’s right leg, polyphalangy (longer-than-normal first digit in a 4-3-2-2′ digit conformation) on the embryo’s left leg, and very mild coloboma of the eye. (**C**) *Moderate expression.* UCD-Coloboma.003 mutant embryo (Z^co^/W) displaying moderate truncation of the limbs, moderate cleft-palate, severe dwarfism, severe exposed viscera, polyphalangy on the both legs, and mild coloboma of the eye. This embryo image was taken from Robb et al. (2011) *Journal of Heredity* 102(2):141–156. (**D**) *Severe expression.* UCD-Coloboma.003 mutant embryo (Z^co^/W) displaying moderate truncation of the limbs, severe cleft-palate, severe dwarfism, severe exposed viscera, oligodactyly (three digits, loss of the hallux) on the embryo’s left leg, polyphalangy on the embryo’s right leg, and severe coloboma of the eye. All individuals are shown at 10 days of embryogenesis.

The individuals utilized for this study were from two genetic lines, the developmental mutant-congenic inbred line UCD-Co.003 and its inbred (F>0.99) parent background line UCD-003 [3], [4]. These two genetic lines are herein referred to as *coloboma* and control, respectively. The *coloboma* (*co* a.k.a *cm*) mutation is sex-linked, recessive (females are the heterogametic sex in birds, Z/W), and an embryonic lethal. Therefore, non-carrier males (Z^+^/Z^+^) and unaffected females (Z^+^/W) are genotypically identical to UCD-003, except for any spontaneous mutations. All affected females (Z^co^/W) are mutants (Figure 1).

### Sample Collection for Fine-Mapping, Validation, and Translocation Assessment

Adult and embryonic blood was collected according to Robb et al. [4] and DNA (with an RNase step) was isolated using the DNeasy® Blood & Tissue kit (Qiagen). DNA samples were isolated from Co.003 normal homozygotes (Z^+^/Z^+^, n = 41), normal heterozygotes (Z^+^/Z^co^, n = 66), and mutant hemizygotes (Z^co^/W, n = 197) and archived for future use as needed. A subset of those samples listed above [n = 28 mutant (Z^co^/W) and 2 control (Z^+^/Z^+^) individuals] was used in the variant validation portion of this study (see below). To test for the putative translocation event (described in [32]), 2 control (Z^+^/Z^+^), 4 heterozygous (Z^+^/Z^co^), and 4 mutant (Z^co^/W; which included the two samples sequenced with the CA/NGS) samples from the archived coloboma DNA were employed.

### SNP-Genotyping, Analysis, and Causative Region Identification for Fine-Mapping

Ten SNPs were selected for their linkage with the *coloboma* trait following an Illumina 60****K SNP array study [4] and used to fine-map the Co.003 CR. Primer sequences are listed in Table S1. The genotypes of the collected samples were determined and/or confirmed at the 10 loci using standard PCR conditions for Phire® Hot Start II DNA Polymerase (Finnzymes) and purified by QIAquick® Spin Kit (Qiagen). Amplicons were sequenced (Davis Sequencing, Davis, CA) using ABI 3730 DNA sequencers (Applied Biosystems) and analyzed for genotype-specific SNP differences. The SNP analysis and CR identification (maximum and minimum CR: CR_max_ and CR_min_ respectively) were defined as previously described [4]. Two *coloboma* mutant samples (co-275F and co-276F) were specifically chosen for CA/NGS based upon their 10 SNP loci genotypes (Table 1).

**Table 1:** SNP fine-mapping by assessment of linked molecular markers in the UCD-Coloboma.003 genetic line resulted in a reduction in the size of the causative region.

				60 K Mutants (Z^co^/W)^A^	60 K Adults (Z^+^/Z^co^)^A^		Post-60 K Fine-Mapped Hemizygous Mutants (Z^co^/W)^B^					Post-60 K Fine-Mapped Heterozygous Adults (Z^+^/Z^co^)^C^				Overall Reduced Region^D^	Normal (Z^+^/Z^+^)^E^
			No. Samples Per Genotype^F^	6	1	3	191^G^	1	1	1^H^	3	1	1	1	63		
SNP ID	SNP	Position^I^	Genotype:														
rs16101051	A/G	20800461		***A***	***AA***	***AA***	***A***	***A***	***A***	***A***	***A***	GA	***AA***	***AA***	GA	***AA***	AA
rs14754601	G/T	20813939		T	GT	GT	T	***G***	***G***	***G***	***G***	GT	***GG***	***GG***	GT	***GG***	GG
rs16761892	A/G	21039041		G	AG	AG	G	G	***A***	***A***	***A***	AG	***AA***	***AA***	AG	***AA***	AA
rs14754985	A/G	21170872		G	AG	AG	G	G	***A***	***A***	***A***	AG	***AA***	***AA***	AG	***AA***	AA
rs14755033	A/G	21219342		A	GA	GA	A	A	***Deletion***	***G***	***G***	GA	***GG***	***GG***	GA	***GG***	GG
rs14755201	C/A	21424689		A	CA	CA	A	A	A	***C***	***C***	CA	***CC***	***CC***	CA	***CC***	CC
GGaluGA349261	G/A	21460798		G	AG	AG	G	G	G	***A***	***A***	AG	***AA***	***AA***	AG	***AA***	AA
rs14755269	G/A	21500736		A	GA	GA	A	A	A	***G***	***G***	GA	***GG***	***GG***	GA	***GG***	GG
rs16101716	A/G	21628290		A	GA	GA	A	A	A	A	***G***	GA	GA	***GG***	GA	***GG***	GG
rs16101791	G/A	21767668		A	GA	GA	A	A	A	A	A	GA	GA	GA	GA	GA	GG
rs14755437	T/C	21798425		T	CT	CT	T	T	T	T	T	CT	CT	CT	CT	CT	CC
GGaluGA349348	A/C	21804207		A	***CC***	CA	A	A	A	A	A	***CC***	CA	CA	CA	***CC***	CC
rs16762348	A/G	21912348		G	***AA***	AG	G	G	G	G	G	***AA***	AG	AG	AG	***AA***	AA
rs14755615	A/G	21990579		G	***AA***	AG	G	G	G	G	G	***AA***	AG	AG	AG	***AA***	AA
rs16102103	A/G	21995335		G	***AA***	AG	G	G	G	G	G	***AA***	AG	AG	AG	***AA***	AA
			CR_min_:^J^	1,194,873	984,486	1,194,873	1,194,873	1,181,395	775,992	494,598	367,044	1,003,745	494,598	367,044	367,044	30,757	
			CR_max_:^J^	1,694,874	1,003,744	1,694,874	1,694,874	1,681,396	1,275,993	994,599	732,077	1,503,746	994,599	732,077	732,077	175,915	

Samples were assessed for their 60****K SNP genotyping pattern to identify recombination events thereby reducing the size of the linked region. Genotypes in bold, italics indicate a region which is no longer linked to the *coloboma* mutation in the particular sample. This region decreased due to recombination and replacement with wildtype (UCD-003) sequence.

A Samples genotyped with Illumina 60****K SNP array [4].

B A total of 197 mutant embryos were collected following the 60****K SNP analysis [4] and these were genotyped using the ten 60****K informative SNPs (Table S1). Two samples, co-275F and co-276F (superscripts ^H^ and ^G^, respectively), were utilized in the CA/NGS technology [32]. Note that co-275F has a minimum and maximum causative region (CR_min_ and CR_max_) of 494,598 and 994,599 bp, respectively.

C A total of 66 heterozygous chicks were hatched post-60****K SNP analysis and were genotyped using the ten 60****K SNPs (Table S1); only three chicks exhibited evidence of recombination events.

D Overall reduced SNP genotype profile based upon compiled genotyping results from both the mutant and heterozygous samples.

E SNP genotype observed in normal, control samples.

F Number of samples analyzed which display each genotype.

G Genotype of sample co-276F, a sample used in the capture array technology.

H Genotype of sample co-275F, a sample used in the capture array technology, which shows a significantly reduced linked region.

I Chromosomal coordinate (bp) of SNP on GGA Z; positions are based on the November 2011 *Gallus gallus* assembly (galGal4).

J Minimum and maximum causative region (candidate gene region) identified for each unique SNP genotype.

### Capture Array Technology, Sequence Variant Identification, and Reference-assisted Assembly

Previously, targeted genomic capture enrichment technology was utilized (SeqWright, Inc.) to sequence the entire 990,267 bp (a.k.a. 990 kb) *coloboma* candidate region on GGA Z (20,813,939-21,804,206 (November 2011, galGal4 assembly) formerly reported as 994,523 bp; (GGA Z: 20,368,747-21,363,270 (May 2006, galGal3 assembly))) [32] as part of a three-mutant sequencing screen. As described by Robb and Delany [32], mutant-specific DNA pools (50 µg total) were sent to SeqWright DNA Technology Services (Houston, TX) for barcoding followed by library production (base pair peak of 150–200), targeted enrichment (for chromosomes 1, 12, and the Z) and SOLiD ™ V3 Plus Platform sequencing (50****bp, single-end sequencing) (Applied Biosystems, Foster City, CA). A total of 3.64 Gbp of data were generated using this technology. Here we described the analysis of the *coloboma* NGS data including SNPs, micro-indels (1–3 nt), macro-indels (4–27 nt), sequence gaps, chromosomal rearrangements, and miRNA binding sites with further assessment of these elements for their potential contribution towards the *coloboma* phenotype.

### Unique Variant Identification and Causative Element Analysis

To refine and reduce the number of potential *coloboma* - causative elements, multiple pairwise-line comparative genomic analyses were utilized (described in [32]). Shared variants were submitted to NCBI (Accessions: ss472340674–ss472343089) and were therein eliminated as possible candidates whereas any variant unique to coloboma alone (i.e., not found within control lines or not previously reported in NCBI and the UCSC genome browsers) was further studied here. Elements found within an exon, UTR, or splice site were validated to differentiate the introgressed-region normal polymorphisms (variants not causative but derived from the original mutant DNA segment, i.e., the source) from the causative element and those elements which remain linked to the *coloboma* mutation (see Table S2 for sequence primers). Table 2 describes the number of each element (e.g., SNP, micro-indel, gap) found at each step of the bioinformatic analyses.

**Table 2:** Coloboma.003 SNPs, micro-indels, and sequence gaps identified in the various linked regions using CA/NGS technology: Number and genomic location.

		Originally Identified in CA^A^				Unique Variants^B^				
CR Identification Methodology	CR Size^C^	SNPs	Insertions^D^	Deletions^D^	Gaps^E^	SNPs	Insertions^D^	Deletions^D^	Gaps^E^	
60****K SNP array	**990,267 nt**	2,500	125	155	525	412	46	65	525	
CA/NGS	**299,860 nt**	895	32	30	186	298	16	17	186	
Fine-mapping	**175,915 nt**	538	27	28	111	245	14	16	111	

A The original number of variants identified after bioinformatics analyses, prior to multiple pairwise-line comparisons to identify unique variants.

B Unique variants are those specific to Co.003 only after multiple pairwise-line genomic comparisons. See Methods for details as to unique variant identification. Note: Shared variants were ruled out as causative towards the *coloboma* mutation and submitted to NCBI (Accession No. ss472340674–ss472343089).

C CR = causative region. 990,268 bp is the CR identified by the 60****K SNP array, which was subsequently utilized for capture array probe creation. 299,860 bp is the size of the CR identified after CA/NGS bioinformatic analysis and further fine-mapping of sample co-275F. 175,915 bp is the CR identified by fine-mapping analysis of recombinant individuals (note that this fine-mapping analysis was ongoing, post-CA/NGS.).

D Insertions and deletions range from 1 to 3 nt in length (within the paper referred to as micro-indels).

E Gaps (≥4 nt DNA which was not captured for sequencing in the CA/NGS) were identified by alignment to the 990,267 nt RJF reference genome [61]. Gaps listed are those found to both be unique to Co.003 as well as those shared across all three mutant congenic lines. Although a gap might be present in all three lines, the missing DNA fragment could contain sequence variation compared to the control.

### Putative Translocation Analysis

In a prior study *de novo* assemblies were compared to identify possible chromosomal (structural) rearrangements, and of five reference-genome assisted assemblies, four indicated a translocation event on GGA Z in the Co.003 congenic line (see Supplemental Figures 1 and 2 in [32]). Here we studied the legitimacy of the putative translocations by designing PCR primers to span the rearranged regions in both control and Co.003 genetic lines. A control PCR amplification was also employed for each translocation assembly primer set to ensure a negative results was not just due to PCR failure. Genomic DNA amplification was carried out as described above. Primer sequence information and results for the putative translocation assessment can be found in Table S3.

**Figure 2:**
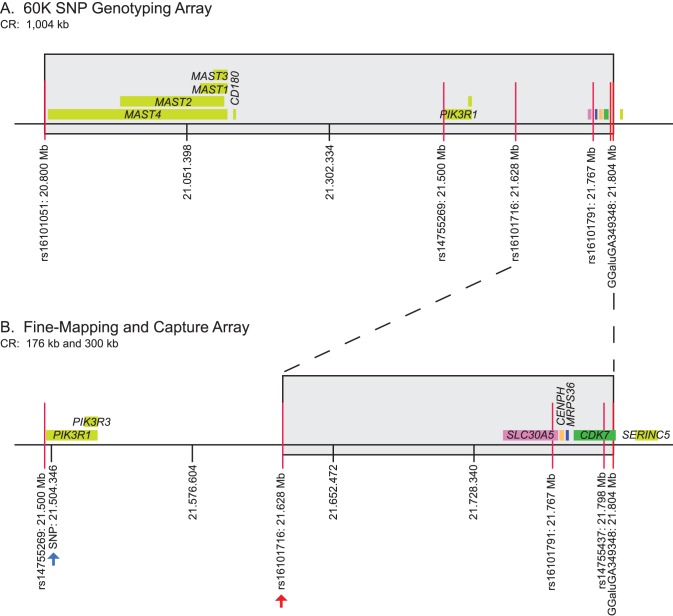
Schematic of causative region size reduction using three technologies. **A.** *Coloboma Z chromosome causative region (CR) identified by the 60* **** *K SNP array:* Six polymorphic SNP markers (red lines) mark the 1.004 Mb causative region (CR_min_) identified by utilization of the Illumina 60****K chicken iSelect SNP genotyping array [4]. SNP markers rs16101051 and GGaluGA349348 denote the boundaries of this region (GGA Z: 20,800,461-21,804,207). Eleven genes (predicted and confirmed) reside within the original 1.004 Mb region (indicated by the gray box). Chromosomal coordinate (bp) on GGA Z are based on the November 2011 *Gallus gallus* assembly (galGal4). **B.** *Coloboma causative region identified by fine-mapping and analysis of the capture array data:* Fine-mapping reduced the causative region to 175,915 bp (aka 176 kb). The following SNP markers designate the boundaries of this region: rs16101716 (red arrow) and GGaluGA349348 (GGA Z: 21,628,290–21,804,207). The 176 kb causative region contains 4 known chicken genes (pink box: *SLC30A5*; orange box: *CENPH*; blue box: *MRPS26*; green box: *CDK7*). Prior to fine-mapping and subsequent reduction to 176 kb, the use of the CA/NGS technology, specifically analysis of sample co-275 sequence data, allowed for a region reduction to 299,860 bp (this boundary is denoted with a blue arrow). Chromosomal coordinate (bp) on GGA Z are based on the November 2011 *Gallus gallus* assembly (galGal4).

### MicroRNA Binding Site Identification in CR Genes

The predicted location of microRNA (miRNA) binding sites were identified using RNAhybrid [33]. The following constraints were utilized: energy cut off (−e) ≤−20; helix constraint (−f) from 2 to 7 (i.e., perfect seed sequence hybridization from nucleotides 2 to 7). Results as to the number of known chicken miRNA-targeted 3′ UTR regions (of known genes in the 176 kb linked region) are listed in Table 3. Additionally, TargetScan 6.0 [34]–[36] was utilized to predict vertebrate-conserved miRNA binding sites (by searching for the conserved 7–8****mer miRNA seed region) encoded within the 3′ UTRs of those genes listed in Table 4. Both the reference genome 3′ UTR gene sequence (obtained from NCBI or UCSC Genome Browser) and Co.003 mutant genomic 3′ UTR gene sequences [32] were crossed checked.

**Table 3:** Variants within the Co.003 Z^co^ causative region are found within predicted chicken miRNA binding sites.

	miRNA Binding Sites	
Gene 3′ UTR	RNAHybrid (Predicted Chicken)	TargetScan (Vertebrate Conserved)^A^
*SLC30A5*	54	2
*CENPH*	282	0
*MRPS36*	60	1
*CDK7*	20^B^	1^B^

A Numbers reflect the number of miRNA binding sites, not the total number of miRNAs shown to bind to that location.

B The 3′ UTR of chicken is not known for this gene therefore the human 3′ UTR was utilized to predict chicken miRNA and vertebrate conserved miRNA binding sites.

**Table 4:** Candidate genes located within the Co.003 GGA Z 175,915 nt CR: gene location, status, function and associated diseases^A^.

Gene	GGA ZLocation^B^	Status ofGene	Identity to Human Protein^B^	Gene Function^C^	Knockout Mouse Phenotype^D^	Affected Anatomical Systems^D^	Associated Diseases^E^	
*SLC30A5*	21744703–21765163	Established	86%	Transports zinc into secretorygranules in pancreatic beta cells;partially regulates zinc homeostasis,maturation of osteoblasts, mastcell activation, mast cell-mediatedallergic reactions, maintenance ofthe cardiac conductionsystem cells.	behavior, mortality/aging	adipose, cardiovascular, growth/size, reduced bone density, skeleton,limbs/digits/tail	***Human*** **:** Associated with several identified QTLs (e.g., body weight,serum cholesterol andapolipoprotein, low densitylipoprotein, serum leptinand glucose levels)	***Mouse*** **:** osteopenia and male-specific sudden cardiac death due to bradyarrhythmia
*CENPH*	21769913–21776085	Established	35%	Component of the active human kinetochore; overexpression induces aneuploidy.	In progress	In progress	***Human*** **:** esophageal and nasopharyngeal carcinomas	***Mouse*** **:** Associated with several identified QTLs (e.g., wound healing)
*MRPS36*	21776248–21776830	Predicted	93%	Role in mitochondrial ribosomal biogenesis.	In progress	In progress	***Human*** **:** Associated with several identified QTLs (e.g., prostate tumor andmyocardial infarctionsusceptibility)	***Mouse*** **:** Type 1 Diabetes
*CDK7*	21786626–21805838	Predicted	80%	Regulators of cell cycle progressionand transcription (required forpolymerase II function).	In progress	In progress	***Human*** **:** Alzheimer’s Disease	***Mouse*** **:** Associated with several identified QTLs (e.g., skeletal muscle weight, glucose homeostasis)

A Although no gene found within this region is a strong candidate for the *coloboma* mutation, based on functionality in other vertebrates alone, several are implicated in mechanisms that may influence the phenotype.

B UCSC genome browser was utilized to identify gene location and percent identity to the human protein. The gene (chicken) coordinate location is based upon the November 2011 *Gallus gallus* assembly (galGal4). Chromosomal synteny was identified in both human Hsa 5 (reference genome February 2009) and mouse Mmu 13 (reference genome July 2007).

C References for gene function are as follows: *SLC30A5* [62]–[68]; *CENPH* [69], [70]; *MRPS36* [71]; and *CDK7* [72]–[75].

D Information for knockout mouse phenotype and affected anatomical systems were obtained from the Mouse Genome Informatics (MGI, http://www.informatics.jax.org/), the International Knockout Mouse Consortium (IKMC, http://www.knockoutmouse.org/), and the Rat Genome Database (RGD) PhysGen Knockouts (http://rgd.mcw.edu/).

E References for the associated disease are as follows: *SLC30A5*: human: http://rgd.mcw.edu/ID=1315825, mouse [76]; CENPH: human [77], [78], mouse: http://rgd.mcw.edu/ID=1312618; MRPS36: human: http://rgd.mcw.edu/ID=1319596, mouse [79]; and CDK7: human [80], mouse: http://rgd.mcw.edu/ID=734280.

### Whole-mount *in situ* Hybridization (ISH)

Expression status of the candidate genes encoded within the *coloboma* fine-mapped CR (175,915 bp) were examined in normal, female coloboma line embryos (Z^+^/W) and mutant female embryos (Z^co^/W) at HH24–HH26, wherein the main morphological features affected in this mutation (e.g. limbs, craniofacial) are sometimes but not always elaborated as yet [37]. A total of 87 newly collected mutant (n = 44) and normal (n = 43) coloboma female embryos (note that this number is not included in the archived coloboma DNA count listed prior) were utilized for ISH. A minimum of four embryos of each group (control and mutant) were tested for every gene. The mutant and normal female embryos used for ISH were identified and selected using the ZZ/ZW (male v female) sexing genotyping, see [32]) and the 10 SNP coloboma genotyping protocol (described above).

Four genes (two confirmed and two predicted) are found within the fine-mapped region. These include solute carrier family 30 member 5 (*SLC30A5*), centromere protein H (*CENPH*), mitochondrial ribosomal protein subunit 36 (*MRPS36*), and cyclin-dependent kinase 7 (*CDK7*). To assess their gene expression during development, RNA probes were created from cDNA (EST) clones (Table S4) acquired from BBSRC ChickEST Database (http://www.chick.manchester.ac.uk/) using methods adapted from Nieto et al. [38]. Protocols for ISH were carried out following Darnell et al. ([39]; http://geisha.arizona.edu/geisha/). ISH embryo images were captured digitally via microscopy (Wild Heerbrugg) by placing embryos on 1.5% agarose-PBS (1×) plates.

## Results

### Mutant Phenotype

*Coloboma* (*co*) is a sex-linked recessive embryonic lethal mutation which affects females. The coloboma syndrome includes dwarfism, craniofacial defects, bilateral facial coloboma, exposed viscera, and absent or greatly reduced extremities due to disruption in cartilage formation [3], [4]. The phenotypic expression of *coloboma* is variable and can be subdivided into three categories: mild, moderate, and severe (see Figure 1A–D). In the majority of cases, the most severe phenotype was displayed in those embryos that died earliest in development (E6–7). Mutants classified as mild (Figure 1B) have a slightly abnormal beak with the proximolateral parts of the maxilla missing and slightly reduced wings and legs; additionally the toe digit pattern can be one of the three conformations described in the subsequent paragraph. Embryos displaying moderate *coloboma* (Figure 1C) expression have phenotypes in between that described for the cases of mild and severe *coloboma*. Severe *coloboma* (Figure 1D) expression includes malformations in the skull and face, with the eyes set forward (due to missing tissue) and the beak and throat reduced such that a cavity in the head is observed. Interestingly, Abbott et al. [3] reported an extreme form with legs either absent or very reduced (described as spikes). However, this extreme leg phenotype was not observed in the 200 mutants phenotyped in this study. Such expression differences are likely due to background genotype, i.e., currently *coloboma* is in the highly inbred (UCD-003) background thereby having different regulatory *cis*- and *trans*-acting factors from the original stock described by Abbott et al. [3]. Table 5 presents the observed cleft-palate expression ratios wherein mild cleft palate is the most prevalent among the mutants.

**Table 5:** Phenotypic variation is observed in the *coloboma* mutants for both cleft-palate and number of digits.

Phenotypic Variation (% observed)							
Cleft Severity^A^			Number of Digits on Feet^B^				
Mild	Moderate	Severe	3 LF, 3RF	4LF, 4RF	4LF, 3RF	3LF, 4RF	5 toes on at least one foot
56.0	21.0	23.0	25.1	37.4	19.8	8.6	9.1

A Cleft-palate severity was recorded for UCD-Co.003 mutant samples (n = 200). In addition to the natural variation of the phenotype, an increase in incubation temperature enhances the severity of the mutation thereby causing an earlier termination of development [4]. See Figure 1 for cleft-palate severity examples.

B Foot digit variation was recorded for UCD-Co.003 mutant samples (n = 187). LF refers to the embryo’s left foot, RF refers to the embryo’s right foot. The number preceding the specific side of the leg (right versus left) indicates the number of digits present.

Mutants can also be characterized by the presence of several different toe digit conformations: (1) a loss of the hallux (inner-most medial digit) displaying a 4-3-2 digit pattern (termed oligodactyly); (2) a longer-than-normal first digit in a 4-3-2-2′ digit pattern (polyphalangy); or (3) an additional pre-axial digit (single-digit duplication) in a 4-3-2-1-2′ digit pattern (pre-axial polydactyly), wherein the duplicated digit is 2′. The variations of digit conformations (4-3-2, 4-3-2-2′, or 4-3-2-1-2′) can be expressed on both feet or in a unilateral fashion with one form on each foot (heterodactyly), with a preference towards 4-3-2 on the mutant embryo’s right leg. See Table 5 for foot digit variation ratios.

### Fine-mapping Reduced the Size of Linked Region and Estimate Co.003 Z Recombination Rate

Robb et al. [4] found a CR_max_ and CR_min_ of 1.504 Mb and 1.004 Mb, respectively (sizes updated using the galGal4 assembly), as defined by 10 SNP loci on the GGA Z associating with the *coloboma* mutation. Following that study, additional mutants were assessed at the 10 SNPs (Table S1) in order to detect recombination events. Analysis of 197 mutants (Z^co^/W) and 66 heterozygotes (Z^+^/Z^co^) led to a reduction of the linked region to 175,915 bp (a.k.a. 176 kb), eliminating 828 kb of genomic DNA and 7 genes (Figure 2). Interestingly, only seven mutants and three heterozygous carriers inherited a recombined chromosome (data not shown). Thus, the recombination rate within the originally-linked 1.004 Mb region on chromosome Z for the Co.003 genetic line was determined to be 0.025–0.030 cM/Mb (LOD score (Z) = 61.4) indicating high linkage disequilibrium in this region. This recombination rate is lower relative to that predicted (1.5–3.5 cM/Mb) for the region by other studies [40], [41].

### Targeted Genomic Capture Enrichment Technology

Although the CA/NGS sequenced a 990,267 bp region found linked to the *coloboma* mutation as identified by Robb et al. [4], the fine-mapping (described above) eliminated a large majority of the region and allowed us to then focus in this study on the reduced region (176 Kb) for the bioinformatics and developmental expression of encoded genes (see below).

#### Reference genome-assisted de novo assembly – translocation identification and validation

Reference genome-assisted *de novo* assembly using Mauve software [42] on the 15.7****M reads generated for the Co.003 genetic line [32] indicated alignments suggesting two putative translocation events involving the mutant chromosome (Z^co^). One larger event consisted of a 53,749 bp DNA fragment translocated (in the 3′ direction) a distance of 258,103 bp on GGA Z^co^. A second, smaller translocation event (7,345 bp) was displaced by 340,463 bp on GGA Z^co^ (in the 3′ direction). Supplemental Figures 1 and 2 in Robb and Delany [32] depict the set of Mauve alignments. Interestingly, in both cases, the translocated DNA fragment was found at GGA Z coordinate 21.59 Mb, just upstream of the *SLC30A5* gene (Table 4).

Here we evaluated each Mauve-predicted translocation to validate the event and consider whether such structural rearrangement could be causative for (1) the low recombination rates calculated for the 1.004 Mb linked region and/or (2) effect the normal expression of a gene(s) within the region thereby possibly contributing to the *coloboma* phenotype. Normal (Z^+^/Z^+^), heterozygous (Z^+^/Z^co^), and mutant (Z^co^/W) samples were all examined for the translocation event using PCR designed to span the rearrangements. In all cases, amplicons were found as predicted by the normal (reference genome alignment) and positive control primers; primers designed to span a putative translocation gave no result (no amplicons) thereby indicating that the Co.003 Z translocation events predicted by Mauve were false (Table S3).

#### Identification of single nucleotide polymorphisms (SNPs) found within the coloboma CR

Some 15.7****M Co.003 CA/NGS reads [32] were mapped to the entire chicken reference genome (May 2006, galGal3 assembly; [11]) and were used to identify SNPs relative to the reference sequence. Upon assessment of the 2,500 SNPs identified within the original 990 kb CR, 2,156 were homozygous and 344 were heterozygous. Any heterozygous SNP was eliminated as being the causative element as the *coloboma* mutation is sex-linked recessive and only females are affected (Z^co^/W) and thus carry only one allele. Of the SNPs found within this region, 1,454 are transition SNPs (A ↔ G or C ↔ T) and 1,046 are transversion SNPs (A ↔ C, A ↔ T, G ↔ C, or G ↔ T), resulting in a transition to transversion ratio of 1.4∶1. This ratio is lower compared to that reported (2.2∶1) in other work [43] and what was observed in two other lines (2.1∶1) used as Z-chromosome controls in the capture array setup [32]. The average SNP density across the sequenced Z^co^ chromosome was 2.4 SNP per kb, approximately two-fold lower than that reported in other domestic chicken lines (5.1–5.8 SNP per kb) [44].

One mutant sample (co-275F) was specifically chosen for use in the CA/NGS technology due to its reduced CR (Table 1). Analysis of the precise breakpoint by the CA/NGS heterozygous SNP genotyping allowed for even further reduction in the size of the *co* linked region to 299,860 bp (a.k.a. 300 kb; GGA Z: 21,504,346–21,804,206) (Figure 2B). Recombinants were identified by analysis of the archived mutant samples, further reducing the linked region to 175,915 nt (a.k.a. 176 kb; GGA Z: 21,628,291–21,804,206). This refined region includes 538 SNPs and 4 genes; 293 of those SNPs were eliminated as causative through multiple pairwise-line comparisons (Table 2).

Of the remaining 245 *coloboma*-unique SNPs within the 176 kb region, 172 are located external to a gene (non-genic) and 90 were found located within a gene inclusive of 77 inside introns, 4 in exons, 4 in 3′ UTRs, and 5 at splice sites (Table 6). The entire set of 245 SNPs were assessed for codon and predicted amino acid changes within the 6 possible reading frames (+1, +2, +3, −1, −2, −3). Reporting only on the first (+1) reading frame (other data not shown), 191 SNPs were synonymous substitutions while 12 SNPs generated nonsense mutations. The SNPs found within the exons, UTRs, and splice sites of known genes (Table 6) were further assessed for causation in the validation portion of this study (see below).

**Table 6:** Location of unique SNPs, micro-indels, and gaps identified by CA/NGS technologies within the candidate genes residing in the 176 kb CR.

	SNP Location^A^			Insertion Location^B^			Deletion Location^C^			Gap Location^D^		
Genes	Exon	Splice Site	Intron	Exon	Splice Site	Intron	Exon	Splice Site	Intron	Exon	Splice Site	Intron
***SLC30A5***	2^E^	2	28	0	0	1	0	3	3	1^F^	2^G^	11
***CENPH***	4^H^	2	12	0	0	0	0	0	2	1^I^	1	2
***MRPS36***	1	1	17	0	0	3	0	0	2	1^J^	0	3
***CDK7***	1	0	20	0	0	2	0	1	3	2^K^	2	8

A All other unique SNPs (n = 155) are non-genic (not found within a gene).

B All other unique insertions (n = 8) are non-genic.

C All other unique deletions (n = 4) are non-genic.

D All other gaps (n = 77) are non-genic.

E One of the unique SNPs identified localizes to a human *SLC30A5* exon; this exon is not present in chicken *SLC30A5* but was still validated.

F This gap spans the 3′ UTR and last exon of *SLC30A5*.

G One of the gaps is located in the promoter region (just upstream (16 nt) of the 5′ UTR) of *SLC30A5*.

H These four SNPs are located within the 3′ UTR of *CENPH*.

I This gap spans the promoter region, 5′ UTR, and the first and second exons of *CENPH*.

J This gap spans the 5′ UTR and first exon of *MRPS36*.

K One of the gaps spans the 5′ UTR and a portion of the first exon of *MRPS36*.

#### Micro-indels (1–3 nts) in the UCD-Coloboma.003 Zco chromosome

Similar to the SNP analysis, the number and location of micro-indels (insertions or deletions relative to the reference genome) of 1 to 3 nucleotides in length were identified. A total of 55 micro-indels (27 insertions, 28 deletions) were identified within the 176 kb region. Of those, 14 and 18 insertions and deletions, respectively, are unique to *coloboma* (Table 2). In total, 12 indels are external to a gene while 20 reside within a gene (15 are intronic and 5 are at splice sites) (Table 6). Upon frame shift analysis, reporting only reading frame +1, 4 of the 32 micro-indels generated nonsynonomous mutations none of which generated a stop codon. The micro-indels exhibited an average distance of 5,578 bp. This value (0.18 micro-indels per kb), calculated for the identified micro-indels in the 176 kb, is lower than that observed on the entire Z chromosome in other chicken breeds (1.44 short indels per kb) [45]. The average micro-indel size was 1.31 bp, three-fold lower than that seen in other chicken breeds (3.7 bp) [45]. It is important to note that this 1.31 bp average micro-indel (≤3 nt) size was calculated for only for the 176 kb region, while the other values were those calculated for the entire Z sex chromosome (3.7 bp average micro-indel size). The micro-indels found within exons, UTRs, and splice sites were further assessed for causation in the validation portion of this study (see below).

#### Identification of gaps within the UCD-Coloboma.003 congenic line

The alignment data was analyzed for the presence of sequence gaps (i.e., putative deletions of ≥4 nts). Within the reduced 176 kb CR there were 111 gaps, with an average size of 52 bp (range: 4 to 634 bp) and an average distance 1,569 bp (range: 51 to 6,926 bp). The gaps within exons, UTRs, and splice sites (Table 2 and 6) were assessed for legitimacy, i.e., true gaps versus sequencing errors, in the validation portion of this study (see below).

#### MicroRNA binding site identification

Predicted miRNA binding sites were identified for both the 176 kb reference genome (Z^+^) and *coloboma* (Z^co^) Z chromosomes. Table 3 displays the number of predicted chicken miRNA binding sites identified within the 3′ UTRs of the genes within the 176 kb region as well as the number of binding sites conserved across vertebrates. Similar to the multiple pairwise-line comparison strategy utilized to eliminate shared sequence variants (SNPs, micro-indels), miRNA binding sites were eliminated as being causative if they shared the same nucleotide sequence with the reference genome. Although a binding site could be normal, a mutant miRNA could cause the *coloboma* phenotype. Further analysis of miRNAs from the region necessitates use of RNA-seq technology.

### Validation of Elements Identified by the Capture Enrichment Technology and Assessment of Causation

The unique SNPs, micro-indels, and sequence gaps associated with the *coloboma* mutation were assessed for their position/coordinate (e.g., exon, intron, etc.) and predicted sequence modification (e.g., synonomous, nonsynonomous). Elements found within exons, UTRs, and splice sites of known and predicted genes across the entire 176 kb sequence were assessed. Additional control (n = 2) and mutant (n = 28) samples were used to verify if a particular variant remained linked to the *coloboma* mutation. Verified SNPs and micro-indels have been submitted to NCBI (Table S2; Accession Numbers: ss475871097–113; ss475871115; ss475871117–28; ss475871130–36; ss475871141–45; ss475871147–69; ss475875322; ss475875324; ss475875337; ss475875345–48; ss475875354; ss550120096–ss550120108).

### Validation of Exonic, UTR, and Splice Site SNPs, Micro-indels, and Gaps

Although the variants within an exon, UTR, or splice site of genes in the 990 kb CR were assessed, only those elements within the 176 kb region are reported here. Table S2 lists additional verified elements (outside the 176 kb), all of which were ruled out thereby confirming the fine-mapping results. A total of 12 SNPs were found within exons, UTRs, or at splice sites. An exon-located SNP was identified in *SLC30A5, MRSP36*, and *CDK7* (Table 6, S2) while four SNPs were located within the 3′ UTR of *CENPH*. Five SNPs were located within a splice site of one of the candidate genes (Table 6, S2). Twenty-eight new mutant samples were used to verify coloboma-linkage at each SNP. All SNPs were validated, i.e., all 12 SNP sequences were found to be legitimate and present in additional mutants. However, the genotyping pattern analysis (haplotype per individual mutant) eliminated all 12 SNPs as being causative of the *coloboma* mutation since at least one mutant individual exhibited the reference genome or the UCD-003 (genetic background) genotype at each SNP locus.

Five micro-indels, more specifically 5 deletions, were found at splice sites of genes within the 176 kb Co.003 GGA Z^co^ sequence (Table 6, S2). The percent validity for these micro-indels was 40% as 2 of the 5 micro-deletions displayed the same genotype as control (UCD-003) samples tested thereby indicating a false-positive micro-indel identification. Upon analysis in additional mutant samples (n = 28) none of the deletions remain linked to the mutation, and therefore none of these can be causative.

Ten gaps were identified within an exon, UTR, or splice site relative to the reference genome (Table 6, S2). In all 10 cases, the gaps were found to be false in both mutant and control samples, i.e., PCR amplification using reference sequence-developed primers produced amplicons with sequence content. This new sequence information was evaluated for variation in mutants v controls. Sequencing identified new sequence content absent from the current assembly as well as new variants (e.g., SNPs and micro-indels). Upon analysis, none of the 10 new sequence variants remain linked to *coloboma*.

### Expression Analysis of Candidate Genes by ISH

**Figure 3:**
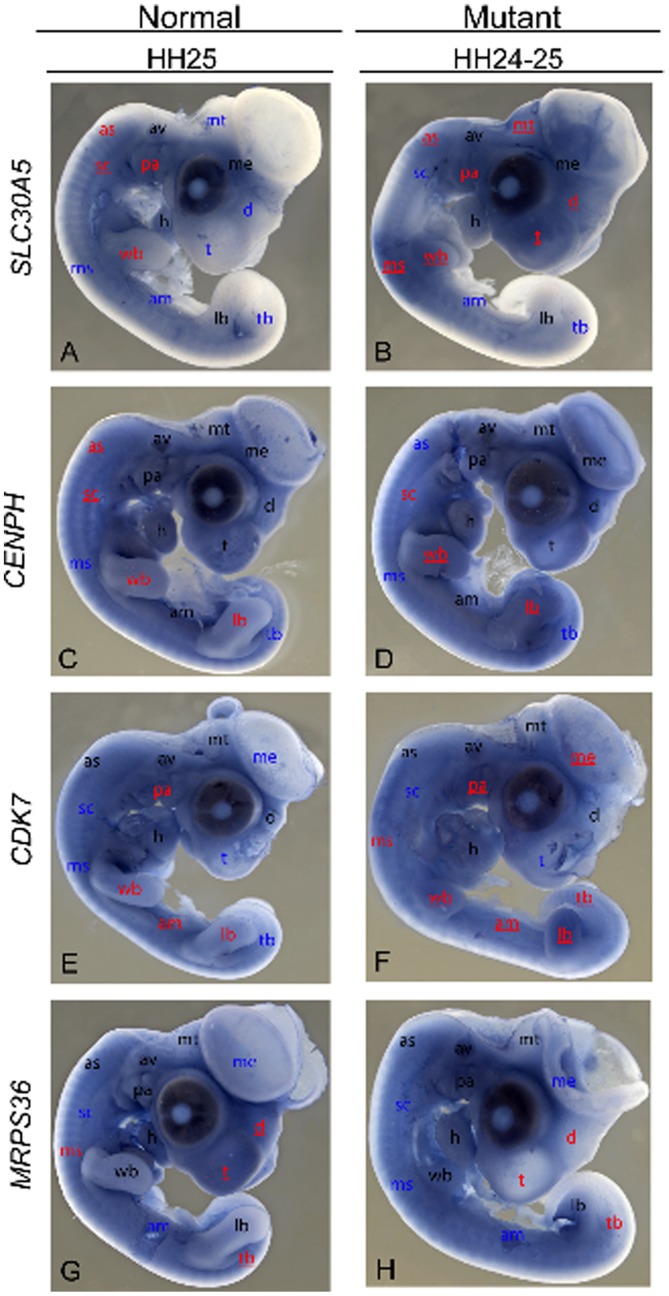
Differential gene expression in normal and mutant embryos from the coloboma line. Localization of chicken *SLC30A5*, *CENPH*, *CDK7, and MRPS36* gene expression in HH stage 24–25 in normal and mutant chicken embryos using whole-mount *in situ* hybridization. See the Results and Discussion sections for expanded explanations. (**A**) Normal coloboma embryo (HH25) with *SLC30A5* expression. (**B**) Mutant *SLC30A5* embryonic expression (HH24) is similar to that of the normal embryo except for increased expression in the telencephalon, pharyngeal arches, wing bud, posterior limb bud, portions of the brain (diencephalon, metencephalon), and the anterior-most and medial somites. (**C**) A representative, normal *CENPH* coloboma embryo. (**D**) Mutant *CENPH* gene expression is similar to that of the normal embryo (C) except for increased expression in the limb buds. (**E**) Normal *CDK7* expression in the coloboma embryo (HH25) is widespread. (**F**) *CDK7* expression within the mutant *coloboma* embryo (HH24) is similarly ubiquitous with an increase in expression in the limb buds, pharyngeal arches, as well as the tail bud (relative to the normal (E)), and the mesencephalon and telencephalon while decreased *CDK7* expression is observed in the abdominal mesoderm. (**G**) The normal coloboma embryo (HH25) displays widespread *MRPS36* expression. (**H**) Similarly, the *coloboma* mutant embryo (HH25) displays ubiquitous expression with decreased levels of expression (relative to the normal coloboma embryo) in the telencephalon, diencephalon and the tail bud. All prominent anatomical locations are labeled (see below for abbreviations). Black indicates that expression levels are the same for both control and mutant embryos. In those embryos where expression is seen in both normal and mutant, the regions are labeled in red and higher expression is indicated by an underline. If a region does not have expression, the area is labeled with blue. Note that for a gene, a region could be marked with blue font in one embryo (no expression, staining at background level) but red-underlined in the other group (expression higher than background). am: abdominal mesoderm; as: anterior somites; av: auditory vesicle; d: diencephalon; h: heart; lb: leg bud; me: mesencephalon; ms: medial somites; mt: metencephalon; pa: pharyngeal arches; sc: spinal cord; t: telencephalon; tb: tail bud; wb: wing bud.

The 176 kb fine-mapped region (galGal4 assembly) encodes four genes. Of these, two genes (*MRPS36* and *CDK7*) are predicted and have chicken ESTs or mRNAs aligned while the other two (*SLC30A5* and *CENPH*) have been confirmed (Table 4). Expression patterns of the four genes were examined for their developmental expression in *coloboma* mutant (Z^co^/W) and coloboma normal (Z^+^/W) female embryos at E5 (HH24 to HH26) to assess their spatial and temporal expression. All anatomical features are at the expected stage of development in the normal coloboma female embryos. However, in the mutant the limbs are often reduced which is to be expected as mutants are dwarfed and display truncated limbs (Figure 1B–D). Largely, expression of the genes at the stages studied can be considered ubiquitous although variation among regions within embryos and between normal and mutants were indicated. Assessment of the mutant embryos at stages of development comparable to the normal (all were from the Co.003 line) indicates three genes as having differential expression levels within *coloboma*-affected tissues: *SLC30A5*, *CDK7* and *CENPH* whereas *MRPS36* showed no evidence of expression differences (see Figure 3). We also examined the gene expression profiles in normal, outbred embryos (HH13– HH25) and the results can be found at the *Gallus* Expression *in situ* Hybridization Analysis (GEISHA) – A chicken embryo Gene Expression Database (http://geisha.arizona.edu/geisha/) [46], [47].

#### SLC30A5

Within the normal developing embryo (HH24), moderate *SLC30A5* expression was observed in the somites, mesencephalon, pharyngeal arches and auditory vesicle (inner ear) while mild *SLC30A5* expression was observed in the heart and in both the posterior wing and leg bud regions (Figure 3A). Figure 3B displays a representative image of *SLC30A5* expression in an HH24 *coloboma* mutant embryo. Strong *SLC30A5* expression is observed in the most anterior somites (1–6) and medial somites (16–21), the pharyngeal arches, posterior wing bud region and in the endoderm of the telencephalon, diencephalon, mesencephalon and metencephalon. Moderate *SLC30A5* expression can be seen in somites 7–19 and 22–27 as well as the distal edge of the wing bud and the posterior leg bud region. Comparing the two representative images for *SLC30A5*, differential expression levels can be seen in the pharyngeal arches, somites, posterior limb bud regions, wing bud, and in several regions of the brain (telencephalon, diencephalon, metencephalon).

#### CENPH

The normal coloboma embryo (HH25) displays strong *CENPH* expression in the entire wing bud, the dorsal surface of the leg bud, the developing heart, abdominal mesoderm, auditory vesicle and pharyngeal arches. Moderate *CENPH* expression can also be seen along the somites, spinal cord, telencephalon, diencephalon, metencephalon, and both wing and leg buds (Figure 3C). Similar to the expression profile observed in the normal coloboma embryo, the mutant displays strong *CENPH* expression throughout the entire body, with an increase of expression in the limbs (compared to the normal embryo) (Figure 3D).

#### CDK7

*CDK7* (Figure 3E) expression at HH24 in normal embryos was observed in the diencephalon, metencephalon, pharyngeal arches, auditory vesicle, and the posterior wing and leg bud region as well as the somites, heart, and abdominal mesoderm. Like the normal coloboma embryo, moderate *CDK7* expression in the mutant (Figure 3F) is seen in the diencephalon, metencephalon, mesencephalon, auditory vesicle, somites, and heart. However, increased expression can be found in the leg and wing buds, pharyngeal arches, mesencephalon, and the tail bud while decreased expression is observed in the abdominal mesoderm.

#### MRPS36

Gene expression of *MRPS36* at HH25 (Figure 3G) in normal embryos shows localization in the head mesenchyme (including: telencephalon, diencephalon, metencephalon), pharyngeal arches, the auditory vesicle, cranial through caudal somites, developing heart and at the distal edge of the wing and leg bud while mild expression is exhibited in the limb buds. Expression of *MRPS36* in mutant embryos is similar to that of the normal embryo except for a minor decrease of expression in the mutant telencephalon, diencephalon, and tail bud (Figure 3H) – these regions are not phenotypically altered in the *coloboma* mutant.

## Discussion

Major advancements have been achieved toward the goal of identifying the causative element for the *coloboma* mutation first described in 1970 [3]. In 2009, a 60K SNP genotyping array established the genomic coordinates of a 990 Kb region and confirmed the sex-linked mode of inheritance on the Z chromosome [4]. The combined power of breeding and SNP-genotyping of mutant (Z^co^/W) and heterozygous (Z^+^/Z^co^) samples identified a recombination event that aided in the reduction of the causative region to 176 kb thereby eliminating 7 genes and 828 kb of DNA sequence.

Advanced genomic technologies (targeted genomic capture enrichment technology) paired with NGS were employed to sequence the entire segregating region associated with *coloboma*. This method offered the opportunity to investigate the region without gene/exon bias (as opposed to exome sequencing) and at high coverage (as opposed to lower coverage obtained through whole genome sequencing) [32]. Bioinformatic analyses identified shared and *coloboma*-specific polymorphic variants as well as previously unknown genomic sequence content, both of which have contributed to the continued refinement of the chicken genome sequence and extant polymorphisms.

The *coloboma*-specific genetic features (putative translocations, 5 micro-indels, 13 SNPs and 10 gaps associated with exons, UTRs and splice sites) were validated in an aim to identify the causative element and unfortunately none of the variations remain linked to the four genes in the region. Given these results, our technology choice was fortuitous in regard to identifying the causative element for this mutation as the CA/NGS data can continue to be mined. Current experiments are focusing on introns and intergenic sequence data as well as functional gene studies.

Given that the validation results showed a lack of linked variants within any coding region of the four genes, we next assessed the gene expression (*MRPS36*, *CDK7*, *CENPH*, *SLC30A5*) in both mutant and normal coloboma embryos in an aim to identify any differences in expression, possibly indicating a role for these genes related to mis- or dys-regulation of expression in the developmental mutant phenotype. *MRPS36* function, gene-associated human/mouse diseases (Table 4), and lack of linked variants paired with its ISH expression profiles (i.e., absence of relevant spatial and temporal expression in *coloboma*-affected anatomical locations in both mutant and normal embryos) do not support *MRPS36* as a candidate gene for *coloboma*.

*CDK7* mutant embryos exhibited increased expression in the limb buds as well as the pharyngeal arches as compared to normal embryos; these anatomical regions are perturbed by the *coloboma* mutation. Note that both mutant and normal embryos showed widespread expression, a profile also conserved in the zebrafish embryo [48], [49]. Functional studies in multiple model organisms, ranging from Drosophila to human, report that this gene is essential for cell division without which there is massive cell cycle arrest and apoptosis [50]–[54]. Furthermore, in *Cdk7* knockout mice, there is early termination at peri-implantation [52]. Although *coloboma* mutant embryos do not survive to post-hatch they do survive for a reasonably long time frame (to day 6–14 (depending on phenotypic severity) of the 21 day incubation period; interestingly, Abbott et al. noted *coloboma* mutant survival to near hatch [3]). Although it seems unlikely, *CDK7* cannot be ruled out at this juncture.

Although *CENPH* expression is widespread in both the normal and mutant coloboma line embryos, increased expression is observed in the limbs of the mutant but appears ubiquitous (and at similar expression levels) in the other mutant-associated anatomical features (Figure 3C–D). As is the case for the other genes, all exon/splice sequence variants within *CENPH* were eliminated. One could conclude from these results alone that *CENPH* is not a high-priority candidate gene for *coloboma*; however, studies in zebrafish suggest further consideration is warranted. *CENPH* may play a role in mitotic failure and aneuploidy [55], which could result in pleiotropic effects relevant to abnormal tissue modeling. Interestingly, *cenph* expression in the stagnant and curly (*stac*) zebrafish mutant showed localization to the craniofacial region, heart, and spinal cord as well as early embryo lethality [55]. The *stac* mutant phenotype includes smaller eyes, vague brain ventricle borders, rough skin, and an upward bent posterior trunk [55]. Thus, we suggest *CENPH* remains a candidate gene. A potential hypothesis warranting further study is that the normal and mutant embryos display a similar *CENPH* mRNA transcript (gene expression) profile but the CENPH protein is truncated or non-functional. Perhaps a variant sequence element upstream or downstream of the gene or within a *CENPH* intron, as could be the case for all of the genes, is generating a miRNA which in turn is binding to the *CENPH* mRNA and results in a lack of protein product (caused by nonsense mediated decay) or a non-functional (truncated) protein (caused by a splice-blocking miRNA). Additional analysis of sequences in the intergenic regions and introns along with protein and functional assays will be necessary to test this and other hypotheses.

Previously we identified *SLC30A5* as a priority candidate gene for the *coloboma* mutation based on comparative analysis of the gene/protein function and knockout mouse studies (heart defects and bone-density loss) [4]. Here we show different expression profiles in the mutant coloboma embryo compared to the normal embryo. Within the normal developing coloboma embryo, only slight/low (or background) expression is observed. The *coloboma* mutant embryo, however, displays increased *SLC30A5* expression in the pharyngeal arches, somites, and posterior wing and leg bud regions perhaps contributing to the craniofacial abnormalities (e.g., coloboma, cleft palate), dwarfism and truncated limbs, and digit malformations (e.g., oligodactyly, polyphalangy, polydactyly), respectively. Although similar to the other candidates, no exon/splice variants within *SLC30A5* remain linked to *coloboma.* It is possible that a non-genic mutation (e.g., in a promoter, chromatin remodeling region, intron, or a copy number variant) is causing the altered expression. Elements (SNPs, micro-indels, gaps) present in the introns and upstream of *SLC30A5* are under investigation.

Advanced sequencing technologies provide massive amounts of data which can lead to causative element identification of inherited developmental mutations. However, the reality of the reverse genetics approach either targeted (in the case of this study) or whole genome sequencing, toward understanding phenotype is extremely complex to decipher. Ultimately the hurdle of bioinformatics, e.g., identification, validation, elimination of artifacts, determination of true polymorphisms, and exploration of natural/normal variation versus causative variation (i.e., “THE element”), must partner with functional studies. As a result of the research reported here and with consideration in other model systems, we postulate three candidates for *coloboma* with an emphasis on one as a high-priority candidate gene. The top priority candidate genes for the coloboma phenotype, based on compiled results (embryonic expression profile, presence of intron and promoter sequence variants (which are currently under investigation), comparative vertebrate biology), are *SLC30A5* and *CENPH*. However, *CDK7* cannot be completely ruled out at this juncture. Current assessment of variants within and around these genes (i.e., within the introns, promoter regions, intergenic regions, etc.) causing a possible alteration in a transcribed miRNA, miRNA binding site, promoter, transcription factor binding site, and transcript splicing, to name a few, are currently being assessed. CNVs (specifically in the case of this mutation, an insertion) are an important consideration as well as they have been described to cause several human mutations and diseases including several craniofacial disorders [56]–[60]. Noteworthy, assessment of known heterozygous (Z^+^/Z^co^) *coloboma* metaphase spreads indicates no observable chromosomal size differences (data not shown) and the validation studies ruled out all putative gaps (aka possible deletions) within the coding sequence of the 4 genes. Thus a CNV (insertion (≤4 nt)) is plausible.

There is much yet to be discovered in regard to normal polymorphisms and mutant variants of both well-studied and novel genes as to their primary sequence and the role of genetic variation as it is elaborated (via protein function) in pathways and networks for developmental processes which impact phenotype. Sequence analysis of the variants (unique mutations) responsible for developmental syndromes (often involving multiple pathways and tissue layers) provide the opportunity to reduce incidence through screening tests and offer a path toward mitigation depending on the causes of the malformation. Expansion of experimental vertebrate models for molecular and cellular analysis is essential to advance the understanding of mechanisms utilized for specific developmental syndromes and diseases in humans. There exist a number of developmental mutations in chicken valuable in contributing to this effort [4] and *coloboma* will undoubtedly be one of those.

## Supporting Information

Table S1 ***Coloboma*** **1 Mb fine-mapping primers used to identify carrier status, causative region size and recombination events.** ^A^Chromosomal location (bp) of SNP on GGA Z; positions are based on the November 2011 *Gallus gallus* assembly (galGal4). ^B^PCR fragment size was determined by three methods: 1) using the UCSC genome browser (http://genome.ucsc.edu/, 2006 *Gallus gallus* assembly (galGal3)), 2) sizing by gel electrophoresis, and 3) DNA sequencing.
(DOCX)

Table S2 ***Coloboma*** **validation primer information for variants within exons, UTRs and splice sites.** Sequence variants found within an exon, UTR or splice site of known genes within the Co.003 linked region (990 kb) were further assessed for linkage to the *co* mutation. (**A**) A total of 14 Co.003 SNPs were found within an exon, UTR, or splice site. (**B**) Six micro-indels were found within an exon, UTR, or splice site within the Co.003 linked region. (**C**) A total of 16 sequence gaps were found within an exon, UTR, or splice site within the 990 kb linked Co.003 region. ^A^Chromosomal location (bp) on GGA Z was assigned based on the November 2011 *Gallus gallus* assembly (galGal4; UCSC genome browser, http://genome.ucsc.edu). ^B^Variant location was obtained through position assessment using the 2011 *Gallus gallus* assembly. ^C^The Primer Reference ID is the internal (Delany Laboratory, UC Davis) tag assigned to that particular variant. Note that cm = co. ^D^PCR fragment size was determined by three methods: 1) using the UCSC genome browser (2006 *Gallus gallus* assembly (galGal3)), 2) sizing by gel electrophoresis, and 3) DNA sequencing. NCBI accession numbers have been assigned to variants (SNPs, indels) identified upon sequencing the capture array gaps. Below outlines the NCBI accession numbers associated with a particular UCD-Co.003 gap sequence variant. ^E^ss475871115; ss475875322; ss475875324 ^F^ss475871117 ^G^ss550120101 ^H^ss475871118–ss475871119 ^I^ss550120102–ss550120103 ^J^ss475871124 ^K^ss475871125–ss475871128; ss475875337 ^L^ss475871130–ss475871133 ^M^ss475871152–ss475871167 ^N^ss550120104–ss550120108 ^O^ss475871168–ss475871169.
(DOCX)

Table S3 **Putative Coloboma.003 translocation assessment: primer and amplicon information, predicted and actual results.** ^A^For the particular Mauve alignment figures, please see [32]. ^B^The Reference genome refers to the normal genome found in the UCSC Genome Browser (http://genome.ucsc.edu). The Translocation genome refers to the Mauve-predicted (putative) translocation and assembly of the coloboma genetic line. ^C^From left to right: the first set of numbers/letters on the left hand size of the primer name refers to the Mauve alignment (e.g., 1–12-Z, Z-1–12, 1-only). Note that primers were designed for only three of the 6 alignments as three of the Mauve alignments were redundant. The “RefG” or “Co” refers to the sequence genome/information from which the primer was designed. The letters after the targeted genome (e.g., P-O, O-G, G-T, P-G, G-O, O-T, R-O, etc.) refers to the section of DNA in each Mauve assembly that primers were designed to flank. For example, under the 1–12-Z Mauve alignment, primers (1–12-Z:Co_P-G) were designed to span the “pink to green” blocks. Lastly, F and R refer to the forward and reverse primers, respectively. See [32] for Mauve alignments. ^D^A dash (–) indicates that no amplification (i.e., no PCR product) is expected. The numbers found in the table refer to the size of the PCR amplicon expected (in basepairs). ^E^Note that the control PCR product produced the proper size amplicon in all samples. Translocation absent indicates that PCR amplicons were visualized based upon the “translocation absent” predictions.
(DOCX)

Table S4 **Chicken EST probes used in whole-mount** ***in situ*** **hybridization: Analysis of UCD-Co.003 candidate gene expression.** ^A^The BBSRC ChickEST database (http://www.chick.manchester.ac.uk) was utilized to identify ESTs for each of the genes. The particular EST sequence can be found at the aforementioned website. ESTs were purchased from Source BioScience UK Limited geneservice (Cambridge, UK), through the BBSRC ChickEST database (http://www.lifesciences.sourcebioscience.com/). Clones were selected on carbenicillin plates (50 µg/mL) prior to growth in LB broth+ carbenicillin (50 µg/mL) and clone purification (using Qiagen’s Plasmid Purification Kit). ESTs were sequenced prior to use in ISH to confirm clone identity. ^B^UCSC genome browser (http://genome.ucsc.edu/) was utilized to identify the location of each gene and the EST percent identity to chicken mRNAs previously identified. The coordinate location is based upon the November 2011 *Gallus gallus* assembly (galGal4). ^C^Each EST clone was inserted and amplified in the pBluescript II KS+ vector, 3.0 kb (Stratagene). The estimated size of each EST was determined through standard restriction enzyme digest (NotI and EcoRI) and subsequent gel electrophoresis. NotI was used to cleave the vector for sense-strand RNA creation using T3 polymerase. Similarly, EcoRI was used, paired with T7 polymerase to generate anti-sense-strand RNA.
(DOCX)
