# Supplementary material for: Defining the Sequence Elements and Candidate Genes for the Coloboma Mutation
Source: PLoS One. 2013 Apr 9;8(4):e60267. doi: 10.1371/journal.pone.0060267 (PMC3621764; doi:10.1371/journal.pone.0060267)
Supplement: Table S1 — Coloboma 1 Mb fine-mapping primers used to identify carrier status, causative region size and recombination events. AChromosomal location (bp) of SNP on GGA Z; positions are based on the November 2011 Gallus gallus assembly (galGal4). BPCR fragment size was determined by three methods: 1) using the UCSC genome browser (http://genome.ucsc.edu/, 2006 Gallus gallus assembly (galGal3)), 2) sizing by gel electrophoresis, and 3) DNA sequencing. (DOCX) [file pone.0060267.s001.docx]

| **SNP** | **Position ^A^** | **Primers (5**' **- 3**'**)** | | **Product Size (bp) ^B^** |
| --- | --- | --- | --- | --- |
|  |  | ***Forward*** | ***Reverse*** |  |
| rs14754601 | 20813939 | TGGGACTGCATTTTCTGTTG | CATGCTCACGCTTCTGCTAA | 222 |
| rs16761892 | 21039041 | CTCAGTTCTAGGGCCTGTCG | CTACTCCCCAGCTGCTCATC | 238 |
| rs14754985 | 21170872 | ACTTCTGAAGCAAAGTCACG | CCTAGTTTTGGCAATTCCTT | 205 |
| rs14755033 | 21219342 | AAGCTTTTTAATTGCAGGTCT | GCGCAGTTTTAGAAGTAGGA | 247 |
| rs14755201 | 21424689 | GAAAGGCTCTAAGGATCCCATT | TGTCTGGGCATGTGGTAAAA | 202 |
| GGaluGA349261 | 21460798 | ACAGCGAGGTATGGATCGTT | TCACCAGAAAGTTGCAAGAAG | 297 |
| rs14755269 | 21500736 | AAAGCAAAGGTTTTTGTTCC | TGGAAATGCCTGCTAAACTA | 161 |
| rs16101716 | 21628290 | AGCAAGAAGCATAAGCAATG | TAGCTGCATAAAAGCACACA | 183 |
| rs16101791 | 21767668 | AAGGCCTGAGAGAAGTGTGC | TCTTTGAACGTGCCTCACAG | 152 |
| rs14755437 | 21798425 | TGTGGGCCAACATAACAAAA | CAAACGAGGCTAATGCACAA | 186 |
