# Supplementary material for: Defining the Sequence Elements and Candidate Genes for the Coloboma Mutation
Source: PLoS One. 2013 Apr 9;8(4):e60267. doi: 10.1371/journal.pone.0060267 (PMC3621764; doi:10.1371/journal.pone.0060267)
Supplement: Table S2 — Coloboma validation primer information for variants within exons, UTRs and splice sites. Sequence variants found within an exon, UTR or splice site of known genes within the Co.003 linked region (990 kb) were further assessed for linkage to the co mutation. (A) A total of 14 Co.003 SNPs were found within an exon, UTR, or splice site. (B) Six micro-indels were found within an exon, UTR, or splice site within the Co.003 linked region. (C) A total of 16 sequence gaps were found within an exon, UTR, or splice site within the 990 kb linked Co.003 region. AChromosomal location (bp) on GGA Z was assigned based on the November 2011 Gallus gallus assembly (galGal4; UCSC genome browser, http://genome.ucsc.edu). BVariant location was obtained through position assessment using the 2011 Gallus gallus assembly. CThe Primer Reference ID is the internal (Delany Laboratory, UC Davis) tag assigned to that particular variant. Note that cm = co. DPCR fragment size was determined by three methods: 1) using the UCSC genome browser (2006 Gallus gallus assembly (galGal3)), 2) sizing by gel electrophoresis, and 3) DNA sequencing. NCBI accession numbers have been assigned to variants (SNPs, indels) identified upon sequencing the capture array gaps. Below outlines the NCBI accession numbers associated with a particular UCD-Co.003 gap sequence variant. Ess475871115; ss475875322; ss475875324 Fss475871117 Gss550120101 Hss475871118–ss475871119 Iss550120102–ss550120103 Jss475871124 Kss475871125–ss475871128; ss475875337 Lss475871130–ss475871133 Mss475871152–ss475871167 Nss550120104–ss550120108 Oss475871168–ss475871169. (DOCX) [file pone.0060267.s002.docx]

| **A. Coloboma.003 SNPs** | | | |  |  |  |  |  |  |
| --- | --- | --- | --- | --- | --- | --- | --- | --- | --- |
| **Chr** | **Coordinat ^A^** | **NCBI Accession No.** | **Reference Genome Allele** | **Mutant SNP (Z^co^/W)** | **Variant Location ^B^** | **Primer Reference ID ^C^** | **Primers (5' - 3')** |  | **PCR Amplicon (bp) ^D^** |
|  |  |  |  |  |  |  | **Forward** | **Reverse** |  |
| Z | 21147478 | ss475871102 | G | A | Splice site of *MAST4,* *MAST1,* *MAST3* | CM-SNP-62 | TCTGCATATTGTTCCCCTCA | TCGGCTCCTGTAGCTGTTTT | 224 |
| Z | 21512623 | ss475871103 | T | A | Splice site of *PIK3R1,* *PIK3R3* | CM-SNP-117 | AGACAAGCGAATGAACAGCA | TTCAGCTTCTTTTGCCGAAC | 210 |
| Z | 21759535 | ss475871104 | C | T | Splice site of *SLC30A5* | CM-SNP-311 | TTAGTGATGGGGCTTTTTGC | TTTAGCTGTGGCAACAGTGG | 246 |
| Z | 21761352 | ss475871105 | T | C | Splice site of *SLC30A5* | CM-SNP-320 | CAAGGCTTGTAGCCAAAACA | CGTGGCTAAAGGCACAGATA | 243 |
| Z | 21761413 | ss475871106 | G | A | Exon of *SLC30A5* | CM-SNP-321 | GGGTATCAGGTGCTGCATTT | CATGGTAAGAATGGCTGTGC | 213 |
| Z | 21771679 | ss475871107 | A | T | Splice site of *CENPH* | CM-SNP-343 | ATGCATTTCCAATGCCAAAG | CAGAAGCCCAGCTTGAACTC | 221 |
| Z | 21775586 | ss475871108 | C | G | 3'UTR of *CENPH* | CM-SNP-351 | GAACATAGCCATGCCACTGA | CGTCAGCCCCTCTGTCTAAG | 206 |
| Z | 21775807 | ss475871109 | A | G | 3'UTR of *CENPH* | CM-SNP-352 | CAAGAATTCATTTCCCCATGA | GCACGTAGTCGTCAGTGCTT | 394 |
| Z | 21775814 | ss475871110 | T | G | 3'UTR of *CENPH* | CM-SNP-353 | TTCATTTCCCCATGAAGTGC | GCAGGTTAGGCACGTAGTCG | 397 |
| Z | 21775940 | ss475871111 | G | A | 3'UTR of *CENPH* | CM-SNP-354 | TGCTCTGAAGTGTGTCTCTGC | GCGCTTTATTTGGGTCGTTA | 225 |
| Z | 21776092 | ss475871112 | G | C | Splice site of *CENPH* | CM-SNP-355 | CGCCTTAAGCACTGACGACT | GCCGATTCATTAATGCAGGT | 215 |
| Z | 21781829 | ss550120096 | C | T | Splice site of *MRPS36* | CM-SNP-372 | CAGAATATTTTCATTGTGCTTTTGA | TGCATTTGAAGCATGAGGTG | 229 |
| Z | 21781909 | ss550120097 | T | C | Exon of *MRPS36* | CM-SNP-373 | AAAAACAGGTTGTGATCAGTTGAG | TTGAGGTAAGATTCTTGCTAATTCC | 293 |
| Z | 21803993 | ss550120098 | T | C | Exon of *CDK7* | CM-SNP-412 | GAATAGTTAGCTTCTGCTGTGGTT | GGCTGTAACTCTAGAACAAGGATT | 250 |
|  |  |  |  |  |  |  |  |  |  |
| **B. Coloboma.003 micro-indels** | | | |  |  |  |  |  |  |
| **Chr** | **Coordinate ^A^** | **NCBI Accession No.** | **Mutant Indel Genotype** |  | **Variant Location ^B^** | **Primer Reference ID ^C^** | **Primers (5' - 3')** |  | **PCR Amplicon (bp) ^D^** |
|  |  |  |  |  |  |  | **Forward** | **Reverse** |  |
| Z | 21154266 | ss475871097 | -AT |  | Exon of *MAST4* | CM-sh-DEL-20 | TGGCTATTAAGGTAAAAAGGAAAAA | CCTTCAAAACTGAATACCTCCAC | 383 |
| Z | 21748002 | ss475871098 | -TT |  | Splice site of *SLC30A5* | CM-sh-DEL-49 | TTGTTTCTTTTGAAACTGTGGAG | TTTCCCAAGTAGCACAGAGGA | 295 |
| Z | 21759995 | ss475871099 | -G |  | Splice site of *SLC30A5* | CM-sh-DEL-51 | TTGAGCCTGAAGCCCTACAT | CACGTCCGTATCTGAAAGACAA | 272 |
| Z | 21760288 | ss475871100 | -A |  | Splice site of *SLC30A5* | CM-sh-DEL-52 | CGGACGTGTAGAAATTCTCTCTG | TGTTGAAAGTCAACCATTTAAAAAGA | 398 |
| Z | 21771035 | ss550120099 | -T |  | Splice site of *CENPH* | CM-sh-DEL-54 | TGCAAGCTGAGAACTGGAGA | GGGGTTTCAATGGTGCTAAA | 297 |
| Z | 21788218 | ss475871101 | -A |  | Splice site of *CDK7* | CM-sh-DEL-59 | CAGATTAAACTGGGCCATCG | ATGCAAGACACAGAGGCACA | 286 |
|  |  |  |  |  |  |  |  |  |  |
| **C. Coloboma.003 sequence gaps** | | | |  |  |  |  |  |  |
| **Chr** | **Gap Range ^A^** | | **Gap Size (bp)** |  | **Gap Location ^B^** | **Primer Reference ID ^C^** | **Primers (5' - 3')** |  | **PCR Amplicon (bp) ^D^** |
|  | **5'** | **3'** |  |  |  |  | **Forward** | **Reverse** |  |
| Z | 20867032 | 20867539 | 508 |  | Exon of *MAST4*, *PLEKHG4* | CM-GAP-27 | AACGAGGTATCTCGCAAACG | CGGATCGCCTTAAACAACAC | 765 |
| Z | 20931616 | 20931650 | 35 |  | Splice site of *MAST4* | CM-GAP-67 ^E^ | TGTTCAAAGTTCTCAGTTTGTGC | GCTGCCTCTTATATTTGAAAGACC | 297 |
| Z | 21149725 | 21149729 | 5 |  | Exon of *MAST4* | CM-GAP-157 ^F^ | TGTTCACGAAAACACAGTCTTG | GCAGCATTCACTTTTTCAGC | 266 |
| Z | 21510082 | 21510093 | 12 |  | Splice site of *PIK3R1*, *PIK3R3* | CM-GAP-342 | CGAGACACTGCCGATGGTA | TTTCCCATCTCGGTGAAATA | 250 |
| Z | 21512607 | 21512609 | 3 |  | Splice site of *PIK3R1*, *PIK3R3* | CM-GAP-343 ^G^ | GCAAGCAGCTGAATATAGAGAGA | CCAAGCCATTCATTCAGCTT | 246 |
| Z | 21516121 | 21516133 | 13 |  | Splice site of *PIK3R1*, *PIK3R3* | CM-GAP-346 ^H^ | CAGTTTTCTTTGTACAGAATATTGAGC | AAAAAGCAAACATAGCGTGGT | 265 |
| Z | 21744490 | 21745015 | 526 |  | 3'UTR and last exon of *SLC30A5* | CM-GAP-485 ^I^ | TCACTTGGATCAACCCTTCC | CACCACTCTCCTCACCCTCT | 829 |
| Z | 21764185 | 21764203 | 19 |  | Splice site of *SLC30A5* | CM-GAP-497 ^J^ | TTTTTGGTGTCATTCTGCAA | TTTGGCAAACAACACTTCTATGA | 232 |
| Z | 21765179 | 21765185 | 7 |  | Splice site of *SLC30A5* | CM-GAP-498 ^K^ | TTGAGTCAAGATGCTTTTGTAAAGA | CAGTCTTCAAGCTACTTTAGGAAGAAA | 281 |
| Z | 21769758 | 21770391 | 634 |  | 5'UTR and first exon of *CENPH* | CM-GAP-503 ^L^ | TGTTCTCCTGCTTCCCTGTC | GCAAAGCAAGAGCGTCAGTA | 866 |
| Z | 21775199 | 21775219 | 21 |  | Splice site of *CENPH* | CM-GAP-506 ^M^ | ATTGGTTTTCTTGCCCATGT | TGGAGATATCTTTCTCGAGCTG | 285 |
| Z | 21776138 | 21776469 | 332 |  | 5'UTR and first exon of *MRPS36* | CM-GAP-506 ^N^ | GTGCCTAACCTGCATTCGAT | TATTCCTCCGCTCTCACGTC | 577 |
| Z | 21786396 | 21786695 | 300 |  | 5'UTR and first exon of *CDK7* | CM-GAP-514 | GTTGGTCCCACCACGAAG | GGGCCTGCTTCAGAGACACT | 592 |
| Z | 21792038 | 21792116 | 79 |  | Splice site of *CDK7* | CM-GAP-518 ^O^ | GCTTTTAGATGCGTTTGGACA | GATAATGCTGCTGGCAGAAAG | 326 |
| Z | 21804098 | 21804105 | 8 |  | Exon of *CDK7* | CM-GAP-524 | TGGTACACCAGACCGAATCC | TTTCACAGACAGAGCTATATGACATT | 283 |
| Z | 21804095 | 21804207 | 113 |  | Splice site of *CDK7* | CM-GAP-525 | TGGTACACCAGACCGAATCC | CCTTGGCTACATAATTGGGAAT | 370 |
