# Supplementary material for: Defining the Sequence Elements and Candidate Genes for the Coloboma Mutation
Source: PLoS One. 2013 Apr 9;8(4):e60267. doi: 10.1371/journal.pone.0060267 (PMC3621764; doi:10.1371/journal.pone.0060267)
Supplement: Table S3 — Putative Coloboma.003 translocation assessment: primer and amplicon information, predicted and actual results. AFor the particular Mauve alignment figures, please see [32]. BThe Reference genome refers to the normal genome found in the UCSC Genome Browser (http://genome.ucsc.edu). The Translocation genome refers to the Mauve-predicted (putative) translocation and assembly of the coloboma genetic line. CFrom left to right: the first set of numbers/letters on the left hand size of the primer name refers to the Mauve alignment (e.g., 1–12-Z, Z-1–12, 1-only). Note that primers were designed for only three of the 6 alignments as three of the Mauve alignments were redundant. The “RefG” or “Co” refers to the sequence genome/information from which the primer was designed. The letters after the targeted genome (e.g., P-O, O-G, G-T, P-G, G-O, O-T, R-O, etc.) refers to the section of DNA in each Mauve assembly that primers were designed to flank. For example, under the 1–12-Z Mauve alignment, primers (1–12-Z:Co_P-G) were designed to span the “pink to green” blocks. Lastly, F and R refer to the forward and reverse primers, respectively. See [32] for Mauve alignments. DA dash (–) indicates that no amplification (i.e., no PCR product) is expected. The numbers found in the table refer to the size of the PCR amplicon expected (in basepairs). ENote that the control PCR product produced the proper size amplicon in all samples. Translocation absent indicates that PCR amplicons were visualized based upon the “translocation absent” predictions. (DOCX) [file pone.0060267.s003.docx]

| **Mauve Alignment** ^A^ | **Primer Sequence Designed From:** ^B^ | **Primer Name** ^C^ | **Sequence (5**' **to 3**'**)** | **Predicted PCR Result** | | | | | | **Actual PCR Result** ^E^ |
| --- | --- | --- | --- | --- | --- | --- | --- | --- | --- | --- |
|  |  |  |  | **Translocation Present** ^D^ | | | **Translocation Absent** ^D^ | | |  |
|  |  |  |  | ***Normal gDNA*** | ***Heterozygote gDNA*** | ***Mutant gDNA*** | ***Normal gDNA*** | ***Heterozygote gDNA*** | ***Mutant gDNA*** |  |
| 1-12-Z | Reference | 1-12-Z:RefG_P-O_F | TGCAGCAAAATTGTATGAGAGAA | 498 | 498 | - | 498 | 498 | 498 | Translocation absent |
|  |  | 1-12-Z:RefG_P-O_R | ACTCCTTGAACTGTACGCTGAGA |  |  |  |  |  |  |  |
|  |  | 1-12-Z:RefG_O-G_F | AAAATGTTATCCCCTGACGTTCT | 621 | 621 | 621 | 621 | 621 | 621 | control PCR |
|  |  | 1-12-Z:RefG_O-G_R | AGGGAGAGAGAGGAGATTGAGAG |  |  |  |  |  |  |  |
|  |  | 1-12-Z:RefG_G-T_F | TGATTTGGAAAGGACATTTGAAG | 593 | 593 | - | 593 | 593 | 593 | Translocation absent |
|  |  | 1-12-Z:RefG_G-T_R | CAGGGAACATGGGTAGATAGTGA |  |  |  |  |  |  |  |
|  | Translocation | 1-12-Z:Co_P-G_F | AAAGTCACAATGTCATAAAGAGTTGG | - | 674 | 674 | - | - | - | Translocation absent |
|  |  | 1-12-Z:Co_P-G_R | CTATGAGGAAAGCTTGAGGGAAC |  |  |  |  |  |  |  |
|  |  | 1-12-Z:Co_G-O_F | TCATTTGATTTGGAAAGGACATT | 299 | 299 | 299 | 299 | 299 | 299 | control PCR |
|  |  | 1-12-Z:Co_G-O_R | TGAAAATGGAGAAGTGAAAGCTC |  |  |  |  |  |  |  |
|  |  | 1-12-Z:Co_O-T_F | TTCTTGGCTGACCATACTTGATT | - | 3064 | 3064 | - | - | - | Translocation absent |
|  |  | 1-12-Z:Co_O-T_R | GACAACCGTGCACATAGCAG |  |  |  |  |  |  |  |
| Z-1-12 | Reference | Z-1-12:RefG_R-O_F | CCTGCATTCAGTATTCCCCAGT | 487 | 487 | - | 487 | 487 | 487 | Translocation absent |
|  |  | Z-1-12:RefG_R-O_R | GGGGAGACCTTGTTGCACTCTA |  |  |  |  |  |  |  |
|  | Translocation | Z-1-12:Co_O-R_F | CATCACCATAAGGCTCTGCTG | - | 400 | 400 | - | - | - | Translocation absent |
|  |  | Z-1-12:Co_O-R_R | GCTCACGCTTCTGCTAAAGACT |  |  |  |  |  |  |  |
|  |  | Z-1-12:Co_R-R_F | CAGCATTTGCAAACTCAATCTC | 270 | 270 | 270 | 270 | 270 | 270 | control PCR |
|  |  | Z-1-12:Co_R-R_R | TCCCTCTGCAAAATAGATGTCA |  |  |  |  |  |  |  |
|  |  | Z-1-12:Co_R-G_F | TGGTTCTGATGTTGCTGAAGAT | - | 579 | 579 | - | - | - | Translocation absent |
|  |  | Z-1-12:Co_R-G_R | GAATTACTGGGCTGAGGTGAAC |  |  |  |  |  |  |  |
| 1-only | Reference | 1-only:RefG_Y-G_F | CGTAGTGCTGTGCATGGAGT | 545 | 545 | - | 545 | 545 | 545 | Translocation absent |
|  |  | 1-only:RefG_Y-G_R | GTGCACTTGGTGAGGAGGAT |  |  |  |  |  |  |  |
|  |  | 1-only:RefG_G-B_F | CTTGCTTCCTTGTGGTCTGG | 686 | 686 | - | 686 | 686 | 686 | Translocation absent |
|  |  | 1-only:RefG_G-B_R | TAGCTCTGGGAGGTGATGCT |  |  |  |  |  |  |  |
|  | Translocation | 1-only:Co_G-Y_F | ATTCATTGAGGCCTGTGGAG | - | 359 | 359 | - | - | - | Translocation absent |
|  |  | 1-only:Co_G-Y_R | GGGAGACCTTGTTGCACTCT |  |  |  |  |  |  |  |
|  |  | 1-only:Co_Y-Y_F | GGAGGTGATCAGGGAGTCAA | 155 | 155 | 155 | 155 | 155 | 155 | control PCR |
|  |  | 1-only:Co_Y-Y_R | CCTTAGGTTGTGCCTTTGGA |  |  |  |  |  |  |  |
|  |  | 1-only:Co_Y-B_F | GCCTGGAAGCTGAAGAGGT | - | 400 | 359 | - | - | - | Translocation absent |
|  |  | 1-only:Co_Y-B_R | GCAGATCAAACAAAGAAGATTTAAGA |  |  |  |  |  |  |  |
