# Supplementary material for: Defining the Sequence Elements and Candidate Genes for the Coloboma Mutation
Source: PLoS One. 2013 Apr 9;8(4):e60267. doi: 10.1371/journal.pone.0060267 (PMC3621764; doi:10.1371/journal.pone.0060267)
Supplement: Table S4 — Chicken EST probes used in whole-mount in situ hybridization: Analysis of UCD-Co.003 candidate gene expression. AThe BBSRC ChickEST database (http://www.chick.manchester.ac.uk) was utilized to identify ESTs for each of the genes. The particular EST sequence can be found at the aforementioned website. ESTs were purchased from Source BioScience UK Limited geneservice (Cambridge, UK), through the BBSRC ChickEST database (http://www.lifesciences.sourcebioscience.com/). Clones were selected on carbenicillin plates (50 µg/mL) prior to growth in LB broth+ carbenicillin (50 µg/mL) and clone purification (using Qiagen’s Plasmid Purification Kit). ESTs were sequenced prior to use in ISH to confirm clone identity. BUCSC genome browser (http://genome.ucsc.edu/) was utilized to identify the location of each gene and the EST percent identity to chicken mRNAs previously identified. The coordinate location is based upon the November 2011 Gallus gallus assembly (galGal4). CEach EST clone was inserted and amplified in the pBluescript II KS+ vector, 3.0 kb (Stratagene). The estimated size of each EST was determined through standard restriction enzyme digest (NotI and EcoRI) and subsequent gel electrophoresis. NotI was used to cleave the vector for sense-strand RNA creation using T3 polymerase. Similarly, EcoRI was used, paired with T7 polymerase to generate anti-sense-strand RNA. (DOCX) [file pone.0060267.s004.docx]

| **Gene** | **EST ID ^A^** | **Clone ID ^A^** | **BLAT Coordinates ^B^** | **Identity to Chicken mRNA ^B^** | **Size (bp) ^C^** |
| --- | --- | --- | --- | --- | --- |
| *SLC30A5* | 603113674F1 | ChEST64c20 | chrZ: 21745163-21757008 | 98.60% | ~2500 |
| *CENPH* | 603154556F1 | ChEST166p14 | chrZ: 21772628-21776035 | 97.10% | ~500 |
| *MRPS36* | 603415439F1 | ChEST339m10 | chrZ: 21776828-21783428 | 99.50% | ~750 |
| *CDK7* | 603603590F1 | ChEST582c23 | chrZ: 21786975-21809241 | 99.80% | ~1000 |
